# Modulation, Shaping and Replicability of UWB Chaotic Radiopulses for Wireless Sensor Applications

**DOI:** 10.3390/s23156864

**Published:** 2023-08-02

**Authors:** Lev V. Kuzmin, Elena V. Efremova, Vadim V. Itskov

**Affiliations:** Kotelnikov Institute of RadioEngineering and Electronics of RAS, Mokhovaya st., 11/7, 125009 Moscow, Russia; efremova@cplire.ru (E.V.E.); vadim.itskov@phystech.edu (V.V.I.)

**Keywords:** UWB signals, UWB chaotic signals, chaotic signal shaping, chaotic signal modulation, UWB chaotic oscillator, chaotic signal replicability

## Abstract

A modulation method is proposed for generating identical UWB chaotic radio pulses using an analog generator of chaotic oscillations. The problem is on the edge of two contradicting requirements: (1) theoretical ability to produce a huge number of various-shape signals, because of high sensitivity to the initial conditions of the generator; (2) the necessity to reproduce oscillations of the same shape both in the receiver and in the transmitter for the implementation of coherent methods of signal processing. The considered method allows us to resolve this contradiction. A single-transistor chaotic oscillator with single power supply and frequency range 100 to 500 MHz is proposed. A mathematical model of the generator (a system of ODEs) was derived. A method of generating chaotic radio pulses with a reproducible shape that could be varied in a manner that is controlled and natural for UWB radio by means of changing the supply voltage of the chaotic oscillator is shown. The mathematical model of the generator is simulated numerically and proves the proposed ideas. The shaping and the replicability of UWB pulses was experimentally proven in an analog domain on a testbed with four instances of the chaotic generator.

## 1. Introduction

The noise-like nature of the chaotic signal and relative simplicity of the devices that produce it are a constant incentive for the design of new options of using chaos. One such option that once gave rise to a rather large wave of interest in chaotic signals was communications. Good reviews of the research done in this area can be found in [1,2,3]. One of the research directions includes ultra-wideband (UWB) wireless communications and UWB wireless sensor networks.

It is necessary to note that, since the end of the 20th century, the use of UWB signals [4,5] in mass-purpose wireless communication systems has the focus of the scientific community. The beginning of research on mass implementation of license-free UWB solutions is associated with the introduction of a spectral mask by the US FCC [5,6] and the subsequent development of a number of international standards for UWB wireless communications, such as IEEE 802.15.3a [7,8] (not adopted in the end), IEEE 802.15.4a (wireless sensor networks) [9], IEEE 802.15.6 (wireless body area networks) [10], and IEEE 802.15.4z [11,12,13]. A number of major consumer electronics manufacturers have already incorporated UWB solutions into their devices [14,15].

The direction of UWB communications, which was new at that time, became a catalyst for the search for a new type of signal that would satisfy the requirements of the spectral mask (distribute the signal energy over the UWB frequency range), provide the required energy per bit, and compensate for the negative effects of wireless channels (multipath propagation, narrowband interference). Chaotic signals, as signals with a large number of freedom degrees and a complex, non-repeatable shape, have become very promising candidates.

One can highlight two areas of application of chaotic signals in wireless solutions: pseudo-random sequences intended for spreading the spectrum and organizing processing gain and carrier oscillations of RF range. With regular as well as with noise-like signals, there is the problem of synthesizing signals with given spectral, cross-, and autocorrelation properties. To date, for regular signals, there are many examples of the synthesis of special waveforms that have the characteristics required for wireless applications. With the introduction of chaotic signals in the field of communications, this task is also being solved.

First of all, one should mention various methods using chaotic signal fragments for transmitting digital information by means of position modulation (PM) [16,17,18,19], frequency modulation of the carrier by a chaotic signal [20], and the use of discrete systems (maps) with chaos to implement frequency hopping [21]. Chaotic sequences are used to modulate the position and sign of UWB ultrashort pulses (USP) [22,23,24,25,26] in the framework of position modulation, where symbols are encoded by USP sequences with chaotic intervals between them. Position modulation with chaotic signals is used in underwater communications [27,28].

It is popular to use chaotic sequences and chaotic signals as signals with a large number of freedom degrees (large base) to organize spectrum spreading and processing gain. For example, chaotic sequences in combination with PPM modulation are considered in [29]. In [30], UWB USPs encode sequences of +1 and −1, which are generated by a logistic map, in particular to whiten the signal spectrum. Spreading sequences obtained using space-time maps with chaos are proposed in [31]. In [32], discrete chaotic sequences spread the spectrum in CDMA. Chaotic spreading sequences in combination with the Fourier transform are considered in [33]. In [34,35,36], an approach is developed to synthesize pulsed chaotic signals that allow coherent reception without the signal copy in the receiver.

Chaotic sequences and chaotic maps are used as a means for synthesizing discrete sequences with given auto- and cross-correlation properties. For example, a method for generating such sequences is proposed in [37]. In [38,39,40], methods are proposed for forming chaotic sequences using chaotic maps, eliminating periodicity (the degradation of chaos) that can occur due to finite machine accuracy. In [41], a similar problem of synthesizing sequences based on the logistic map and the Fibonacci sequence is considered in view of eliminating periodicity in signals generated by digital systems. A method for generating pseudo-random chaotic sequences using FPGA is described in [42]. In [43], the problem of synthesizing spreading sequences that are superior in their characteristics to Gold sequences is posed and solved. Statistical properties of discrete sequences obtained in a logistic map with chaos are improved in [44]. A method for synthesizing spreading sequences using chaotic maps for a satellite navigation system is proposed in [45].

Certain aspects of the optimal detection of chaotic sequences due to processing gain are being investigated. In [46], the problem of restoring the chaotic map used for the chaotic PM is solved with the Kalman filter. A method for forming chaotic sequences is proposed in [47], which, according to the validation criterion of their detection in a noisy channel, should be better than Gold code. In [48], aspects of detecting discrete chaotic sequences are analyzed, and criteria for their detection are established. The issues of blind detection of a chaotic signal using a neural network are investigated in [49].

Finally, one must mention potential applications of chaotic sequences in the problems of spatial signal separation and beam-forming in promising wireless systems. MIMO-radars with large antenna spacing are discussed in [50]. The problem of forming narrow radiation patterns (uncertainty functions) for radars on chaotic signals is stated in [51]. In [52], the problem of directional communication by means of spatial-frequency separation using chaotic sequences is discussed.

In addition to the digital methods for generating fragments of chaotic signals described above, methods for their analog formation are also being developed. For example, a method for generating a pulse train with chaotic intervals between pulses is proposed in [53], the chaos being produced by the Lorentz system. A microchip of a Gaussian pulse generator for UWB impulse radio is proposed in [54]. A generator of chaotic radio pulse trains on a klystron is described in [55], and a generator of UWB chaotic signals of the microwave range based on a TWT with 22.5 W peak power is described by the same authors in [56].

The formation of signals of a given shape, both chaotic and regular, using analog generators, of course, has also been analyzed in the literature. For example, refs. [57,58] suggest UWB pulse shaping methods for Gaussian and single-cycle ultrashort pulses. The chaotic shaping filter is discussed in articles [59,60] for wireless and radar applications. A new method for controlling a chaotic trajectory, which solves the problem of the coexistence of radar and communication systems, is also proposed in [61]. Moreover, the formation of chaotic signals is considered not only in radio communications, but also in hydroacoustics [62] and in wireless power transmission applications [63].

As can be seen from the above short review, the synthesis of chaotic signals with predictable characteristics is mainly concentrated in the digital domain. At the same time, the synthesis of pulsed chaotic signals with the help of analog systems and analog generators remains behind the scenes. From an engineering and practical point of view, this is more convenient, since there is no need to transfer a digital signal to a radio frequency.

Both digital and analog generators of chaotic oscillations have a fundamental feature: their sensitivity to initial conditions. If, however, for digital chaotic systems, this circumstance does not prevent the synthesis of signals with a given shape and characteristics due to the possibility of controlling the initial conditions digitally, then for analog systems with chaos, this is a significant obstacle that prevents the controlled (predictable) formation of chaotic signals of various waveforms and their reproducibility (replication) both by the same generator of chaotic oscillations and by different copies of the generator. The ability to reproduce the form of carrier oscillations in different devices is necessary in view of coherent methods of transmitting and receiving chaotic signals, in which analog sources of chaotic oscillations participate in one form or another.

As was previously shown [64,65,66,67], when forming chaotic radio pulses, it is possible to reproduce (within certain limits) the initial part of the chaotic signal. In these works, a piecewise linear mathematical model of a chaos generator with a piecewise linear current-voltage characteristic of a transistor was considered. On the basis of this model, the possibility of obtaining chaotic radio pulses from the same chaos generator with coinciding initial sections was theoretically established. A simple experiment was also set up in which this phenomenon was visualized. At the same time, there remains a need for additional research that could show how to reproduce this phenomenon in other types of generators from an engineering point of view, which is very important for practical applications.

In this paper, these results were used to further develop the chaotic radio pulse formation methods that allow repetition of the pulse shape and the shape control. Chaotic radio pulses are used in direct chaotic communications (DCC) in the microwave frequency range [68,69], in personal and local-area wireless sensor networks [70,71,72,73,74,75], and in active sensor networks. Therefore, the results of this research could be applied in practice in already existing wireless communication systems using UWB chaotic oscillations, where only an incoherent receiver of radio signals has been used so far.

The purpose and novelty of this work is to experimentally show that it is possible to solve the problem of forming analog pulse signals of various shapes, which can be repeated from pulse to pulse both for the same generator and for different identically designed instances of the generator.

The article is organized as follows. First, the structure and mathematical model of a chaos generator are described; chaotic oscillations are shown in the model, and the principle of forming chaotic radio pulses with a repeating shape is explained. Then, a testbed with four different instances of the generator is described. In the Section 3, generation of similar chaotic radio pulses is shown both in the numerical model and in the experimental testbed. Experimental data for similar-shape chaotic radio pulses of different generators are presented, as well as the data on the formation of chaotic radio pulses of various shapes.

## 2. Materials and Methods

The method for forming close-in-shape chaotic radio pulses was studied by numerical simulation of a dynamic system that described a model of a chaos generator and by means of experimental prototyping, for which purpose four instances of identical-design generators were mounted on a testbed. Modeling does not allow us to assess all the factors that affect the shape of chaotic oscillations due to the sensitivity of the chaotic generation to initial conditions and to the inaccuracy of setting the parameters of electronic components of the generator. The influence of such factors is difficult to analyze in a numerical simulation, so an experiment is the best way to show their real impact.

### 2.1. Model of Chaotic Generator

**Figure 1:**
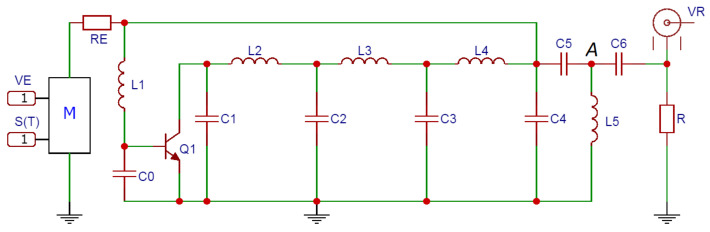
Schematic of chaos generator: $V_{E}$—power supply amplitude, s(t)—sequence of video pulses, $V_{R}$—generator output signal, *M*—power supply modulator.

The generator model described here (Figure 1) belongs to a class of single-transistor oscillator models. Theoretical and experimental approaches to the design of such oscillators have been developed over a number of years. The starting point was the Colpitts oscillator, the chaotic modes of which were shown in [76]. This type of chaotic oscillator has been popular and is still being actively studied [77,78,79].

The schematic of the generator was a product of a number of papers [80,81,82] that resulted in chaotic oscillation sources suitable for engineering applications in the field of wireless communications [70,71,72,73,74]. Such generators provide chaotic oscillations in the prescribed frequency band and have wide zones of chaotic generation in the parameter space, which ensures their stable operation in the chaotic mode in both the case of a natural parameter spread of standard radio components and the case of the power supply instability of real technical systems.

In this paper, we propose a new generator that has a high-dimensional (12th order) frequency-selective system that allows us to form chaotic oscillations in the frequency range 100 to 500 MHz, with a flat spectrum envelope within this band. This frequency range was taken to conveniently analyze the results using an oscilloscope with a 500 MHz input bandwidth and a 2.5 GHz sampling rate. The generator has one tap of power supply, with which the operation mode is controlled (Figure 1).

According to the method of [64,65,66], UWB chaotic radio pulses in the generators of this class are formed by turning the power ($V_{E}$) on and off at specified time moments. The power itself is turned on and off by an external video signal s(t). The generator operation mode is determined by the amplitude $V_{E}$ of power pulses. The oscillations are excited when the base–emitter junction of the transistor opens due to the applied voltage.

The mathematical model of the generator (derived from the Kirchhoff laws) is described by the following system of equations (see Appendix A and Appendix B):

(1) $$\left\{\begin{array}{cc} \frac{L_{5}}{\alpha R}{\dot{i}}_{5} & =v_{a}\\ \frac{L_{1}}{\alpha R}{\dot{i}}_{1} & =v_{4}-v_{0}\\ \frac{RC_{0}}{\alpha }{\dot{v}}_{0} & =i_{1}-\frac{1}{\beta }i_{c}\left(v_{0}\right)\\ \frac{RC_{1}}{\alpha }{\dot{v}}_{1} & =i_{2}-i_{c}\left(v_{0}\right)\\ \frac{RC_{2}}{\alpha }{\dot{v}}_{2} & =i_{3}-i_{2}\\ \frac{RC_{3}}{\alpha }{\dot{v}}_{3} & =i_{4}-i_{3}\\ \frac{L_{2}}{\alpha R}{\dot{i}}_{2} & =v_{2}-v_{1}-\frac{R_{2}}{R}i_{2}\\ \frac{L_{3}}{\alpha R}{\dot{i}}_{3} & =v_{3}-v_{2}-\frac{R_{3}}{R}i_{3}\\ \frac{L_{4}}{\alpha R}{\dot{i}}_{4} & =v_{4}-v_{3}-\frac{R_{4}}{R}i_{4}\\ \frac{RC_{4}}{\alpha }{\dot{v}}_{4} & =\frac{R}{R_{E}}\left(v_{E}s\left(t\right)-v_{4}\right)-i_{1}-i_{4}-i_{5}-v_{R}\\ \frac{RC_{5}}{\alpha }{\dot{v}}_{a} & =\frac{RC_{5}}{\alpha }{\dot{v}}_{4}-i_{5}-v_{R}\\ \frac{RC_{6}}{\alpha }{\dot{v}}_{R} & =\frac{RC_{6}}{\alpha }{\dot{v}}_{a}-v_{R} \end{array}\right.$$

where the dimensional time *t* is coupled with the nondimensional time $t_{d}$ as $t=t_{d}\alpha$, $\alpha =2\pi \sqrt{L_{5}C_{5}}$; dimensional voltage *V* with nondimensional *v*: $V=V_{T}v$; dimensional current *J* with nondimensional *i*: $J=(V_{T}/R)i$, where $V_{T}=0.75$ Â is the barrier potential of the transistor, R=50 Ohm. Here, $v_{k}$, k=0…4 are voltages over capacitors $C_{k}$, respectively; $v_{a}$, $v_{R}$ are voltages in point *A* and over the load *R*, respectively; $i_{k}$, k=1…5 are currents through inductances $L_{i}$. $V_{E}$ is power supply voltage, and s(t) is a sequence of rectangular video pulses with unit amplitude.

The current–voltage response of the transistor, which relays the current through the collector to the voltage at the base–emitter junction, is described by the Ebers–Moll model: $J_{c}\left(V_{0}\right)=I_{0}\left(\exp\left(V_{0}/V_{n}\right)-1\right)$. The dimensionless form of the equation is:

(2) $$i_{c}\left(v_{0}\right)=i_{0}\left(\exp\left(v_{0}\frac{V_{T}}{V_{n}}\right)-1\right)$$

where $I_{0}=0.22$ fA is the reverse saturation current of the base–emitter junction, $I_{0}=i_{0}\frac{V_{T}}{R}$, $V_{n}=25.3$ mV.

Let system (1) have an equilibrium state $(v_{0}^{\left(0\right)}$, $v_{1}^{\left(0\right)}$, $v_{2}^{\left(0\right)}$, $v_{3}^{\left(0\right)}$, $v_{4}^{\left(0\right)}$, $v_{a}^{\left(0\right)}$, $v_{R}^{\left(0\right)}$, $i_{1}^{\left(0\right)}$, $i_{2}^{\left(0\right)}$, $i_{3}^{\left(0\right)}$, $i_{4}^{\left(0\right)}$, $i_{5}^{\left(0\right)})$, in which, by definition, the derivatives of currents and voltages are equal to zero: ${\dot{v}}_{k}=0$, k=0…4, ${\dot{v}}_{a}=0$, ${\dot{v}}_{R}=0$, ${\dot{i}}_{k}=0$, k=1…5. Then, after the necessary transformations, we obtain:

(3) $$\left\{\begin{array}{cc} \frac{R}{R_{E}}\left(v_{E}-v_{0}^{\left(0\right)}\right) & =\left(1+\frac{1}{\beta }\right)i_{c}\left(v_{0}^{\left(0\right)}\right)\\ v_{1}^{\left(0\right)} & =v_{0}^{\left(0\right)}-\frac{R_{2}+R_{3}+R_{4}}{R}i_{c}\left(v_{0}^{\left(0\right)}\right)\\ v_{2}^{\left(0\right)} & =v_{0}^{\left(0\right)}-\frac{R_{3}+R_{4}}{R}i_{c}\left(v_{0}^{\left(0\right)}\right)\\ v_{3}^{\left(0\right)} & =v_{0}^{\left(0\right)}-\frac{R_{4}}{R}i_{c}\left(v_{0}^{\left(0\right)}\right)\\ v_{4}^{\left(0\right)} & =v_{0}^{\left(0\right)}\\ v_{a}^{\left(0\right)} & =0\\ v_{R}^{\left(0\right)} & =0\\ i_{1}^{\left(0\right)} & =\frac{1}{\beta }i_{c}\left(v_{0}^{\left(0\right)}\right)\\ i_{2}^{\left(0\right)} & =i_{c}\left(v_{0}^{\left(0\right)}\right)\\ i_{3}^{\left(0\right)} & =i_{c}\left(v_{0}^{\left(0\right)}\right)\\ i_{4}^{\left(0\right)} & =i_{c}\left(v_{0}^{\left(0\right)}\right)\\ i_{5}^{\left(0\right)} & =0 \end{array}\right.$$

From the radio engineering point of view, the equilibrium state (3) is equivalent to the stationary currents and voltages established in the system (1) under the constant supply voltage $V_{E}$: the currents through the inductances are equal to zero, the difference of potentials at point *A* and load *R* are zero, and the voltages on the capacitances $C_{0}$, $C_{1}$, $C_{2}$, $C_{3}$, $C_{4}$, $C_{5}$, $C_{6}$ are established as a result of dividing the supply voltage $V_{E}$ by resistances $R_{E}$, $R_{1}\ldots R_{4}$.

The solution to system (3) is unique, so system (1) has a simple equilibrium state. The values of the variables defining state (3) are found from the solution of the first nonlinear equation of the system (3), which is unique:

(4) $$\begin{array}{cc} \frac{R}{R_{E}}\left(v_{E}-v_{0}^{\left(0\right)}\right) & =\left(1+\frac{1}{\beta }\right)i_{c}\left(v_{0}^{\left(0\right)}\right). \end{array}$$

On the dynamics of a 12-dimensional system (1) of differential equations, only general qualitative judgments can be made. Namely, its single equilibrium state is determined by the solution of nonlinear Equation (4). The dependence of (4) $v_{0}^{\left(0\right)}$ on $v_{E}$ is monotonic, i.e., the more $v_{E}$, the more is $v_{0}^{\left(0\right)}$. When $v_{0}^{\left(0\right)}$ exceeds the threshold value for unlocking the base–emitter junction of the transistor, the conditions for excitation of oscillations are fulfilled. This means that, at a certain value of $v_{E}$, equilibrium state (3) loses stability.

The dynamics of the model system were numerically studied for parameter values $L_{1}=55$ nH; $L_{2}=110$ nH; $L_{3}=47$ nH; $L_{4}=12$ nH; $L_{5}=33$ nH; $C_{0}=0.4$ pF; $C_{1}=1.5$ pF; $C_{2}=10$ pF; $C_{3}=15$ pF; $C_{4}=15$ pF; $C_{5}=5$ pF; $C_{6}=10$ pF; $R_{E}=150$ Ohm; R=50 Ohm; $R_{1}=25$ Ohm; $R_{2}=25$ Ohm; $R_{3}=25$ Ohm; $R_{4}=25$ Ohm; $R_{5}=25$ Ohm; and β=200.

**Figure 2:**
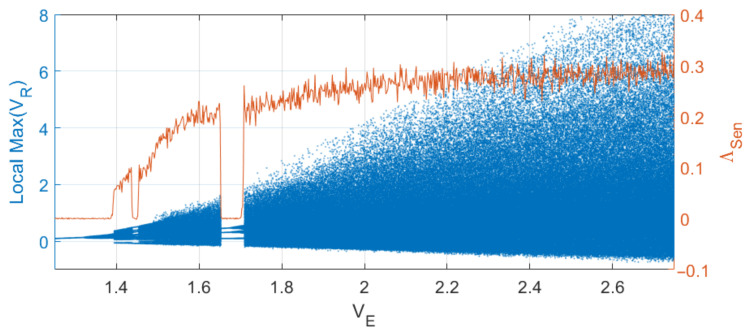
Bifurcation diagram (blue, left-hand scale) and the largest Lyapunov exponent (red, right-hand scale) of system (1) as a function of power supply voltage $V_{E}$.

The bifurcation diagram of the generator is shown in Figure 2. For the given parameter values, chaotic oscillation modes take place in a wide range of supply voltage $V_{E}$. The chaotic nature of the oscillations is confirmed by the analysis of the largest Lyapunov exponent, which is shown in Figure 2 as a red line. As can be seen, the largest Lyapunov exponent is positive in a wide range of the bifurcation parameter $V_{E}$.

**Figure 3:**
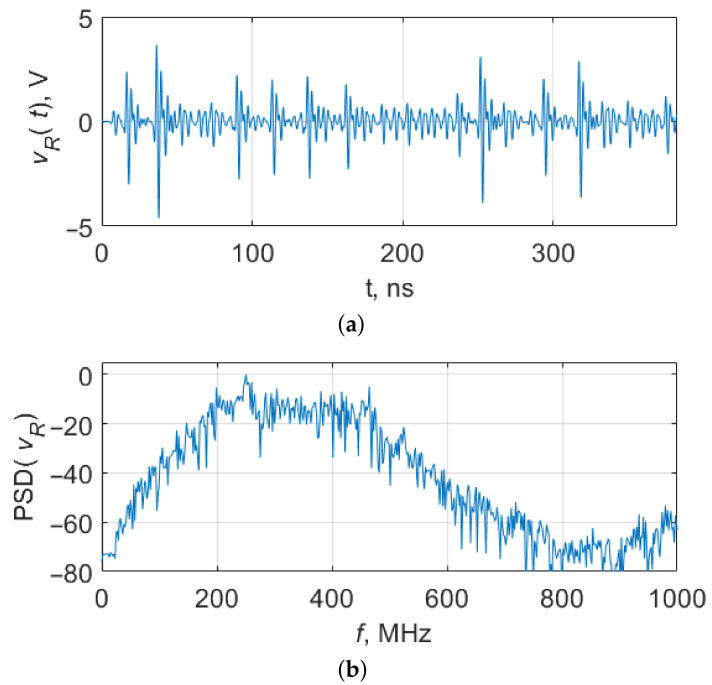
(**a**) A fragment of chaotic oscillations of system (1) at $V_{E}=2.3$ V and (**b**) power spectrum.

An example of the oscillations and their power spectrum for a model value $V_{E}=2.3$ V is given in Figure 3.

### 2.2. Generator Testbed

**Figure 4:**
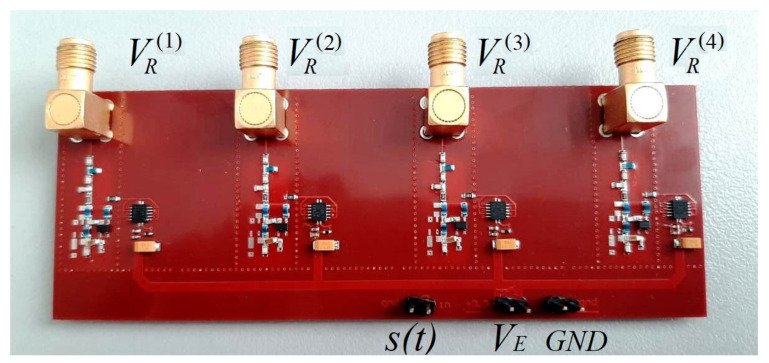
Testbed of four identical chaos generators (Figure 1): s(t)—input for control signal, $V_{E}$—input for common power supply, GND—ground, $V_{R}^{\left(1\right)}$, $V_{R}^{\left(2\right)}$, $V_{R}^{\left(3\right)}$, $V_{R}^{\left(4\right)}$—microwave outputs of the generators.

The concept of the generator presented in the paper was verified experimentally on a testbed, which included four identical instances of the chaotic oscillation generator mounted on a printed circuit board with same-type radio components (Figure 4). The component ratings were within 2% accuracy. We used general-purpose, commercially available radio components. The topology of the generators was identical.

The schematic of each generator corresponds to Figure 1. The testbed contains no passive resistances of the inductances as separate radio-elements ($R_{1}=0$ Ohm, $R_{2}=0$ Ohm, $R_{3}=0$ Ohm, $R_{4}=0$ Ohm, $R_{5}=0$ Ohm). In the numerical model, these resistances are necessary to simulate the energy dissipation, which is inevitable in a real device, and which cannot be fully taken into account in the mathematical model (1) at the level of Kirchhoff’s laws. In the testbed, these resistances are not needed as separate elements, since the inductances have their own passive resistances and since there is natural dissipation and nonlinearity of a real transistor, which ensures limited oscillation amplitude.

The generators are supplied through a common power input $V_{E}$, from which the power is fed to each generator. The length of the power conductors connecting the common power input with the power input of each separate generator is made equal to ensure that all the generators can be turned on/off simultaneously. Each generator is separately powered by switches controlled by the s(t) signal, so that generator $V_{E}$ is powered if s(t)=1 and not powered if s(t)=0. Varying the supply voltage $V_{E}$ allows us to vary the oscillation mode of the generators.

The outputs $V_{R}^{\left(1\right)}$, $V_{R}^{\left(2\right)}$, $V_{R}^{\left(3\right)}$, and $V_{R}^{\left(4\right)}$ of the generators are connected to a four-channel oscilloscope by coaxial cables.

## 3. Results

Below are the results on forming close-in-shape chaotic radio pulses in the model of the generator and in the experimental layout.

### 3.1. Formation of Close-in-Shape Chaotic Radio Pulses in the Mathematical Model of the Generator

The mechanism and the method for generating similarly shaped pulses considered in this paper are based on the modulation of the generator power supply by a video signal [64,65,66].

**Figure 5:**
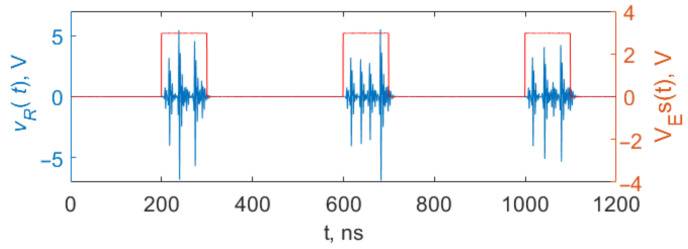
A sequence of chaotic radio pulses with duration of 100 ns, generated in model (1) under the action of video pulses s(t) with amplitude $V_{E}$.

When modulated by video pulses that abruptly turn the power on, the generator is abruptly transferred from rest mode to chaotic generation mode (Figure 5). In rest mode, the dynamic system is in a stable equilibrium state. When the system jumps from rest mode to generation mode, the oscillations start from approximately the same initial conditions (the fixed point of the dynamic system (3)) every time, which ensures the signal reproducibility in the initial section of the oscillations. Consequently, the system generates chaotic radio pulses, the initial sections of which are close to each other. However, over time, the signal shapes become different as the phase trajectories diverge, since they start from initial conditions that in reality are slightly different.

Numerical calculations show that, like the system described in [64,65,66], a similar situation occurs in the system proposed in this paper, despite the significant differences between these systems (different structure, higher dimensionality, and different nonlinearity type: exponential instead of piecewise linear). If the duration of the interpulse interval is large enough for the system to relax to a stable equilibrium state, where it starts upon the arrival of the next pulse, the dynamic system forms chaotic radio pulses with identical initial sections.

**Figure 6:**
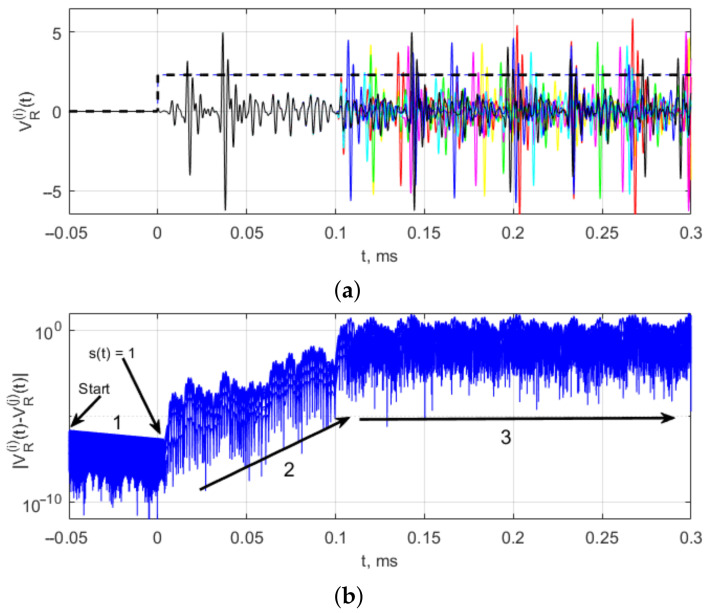
(**a**) The shape of the initial sections of seven differently colored pulses (black, red, blue, yellow, green, blue, magenta) in model (1) and (**b**) pairwise differences between seven pulses (1, 2 and 3 are initial sections of chaotic radio pulses.

The results of simulation of the chaotic radio pulse generation by system (1) are shown in Figure 6a, which gives the initial fragments of seven pulses. Figure 6b shows the absolute magnitude of the pairwise differences between the waveforms of these seven pulses, $D_{k}\left(t\right)=\left|V_{R}^{\left(i\right)}-V_{R}^{\left(j\right)}\right|$, i,j=1,3,…,7, i≠j, on log scale.

The difference signal on a logarithmic scale visualizes the evolution of the trajectories after the start from the initial conditions. At the initial moment (in Figure 6, −0.05 ms), the initial conditions are set at random, and the generator is not powered. In this case, the trajectory, left to itself, tends to a fixed point, a stable equilibrium state, which is expressed as a decrease in the absolute difference between the trajectories (in Figure 6a declining section 1). This process lasts up to the zero moment, when the power-on switching is initiated (a video pulse arrives), the fixed point becomes unstable, oscillations occur, and, in the course of further evolution, the trajectories diverge, with the difference between them increasing (inclining section 2) up to values corresponding to the oscillation amplitude on the attractor, which on average is characterized by a constant upper bound of the difference (horizontal section 3). Thus, the possibility of generating identical chaotic radio pulses in a chaos generator is also confirmed for model (1). As can be easily seen in Figure 6a, the initial shape is reproduced from pulse to pulse.

### 3.2. Variation of the Oscillation Shape in the Mathematical Model of the Generator

The approach to the formation of pulses with different shapes and the reproducibility of this shape from pulse to pulse are based on two complementary properties of system (1): reproduction of close-in-shape oscillations in the initial stage and exponential instability of the chaotic trajectory with respect to perturbations. Combining these two properties allows us to form pulses having a different shape than the initial section, which can be reproduced from pulse to pulse, or to form identical pulses of different shapes, if the length of the power supply pulse does not exceed the duration of the repeating section of the trajectory. Below we describe one of the possible ways of implementing the proposed approach to obtaining oscillations of different shapes. The idea behind the method is that different values of the supply voltage are fed to the chaos generator, depending on the required pulse shape. In this case, oscillations with different shapes are excited, which corresponds to the system transition from the rest state to one or another mode of chaotic oscillations.

**Figure 7:**
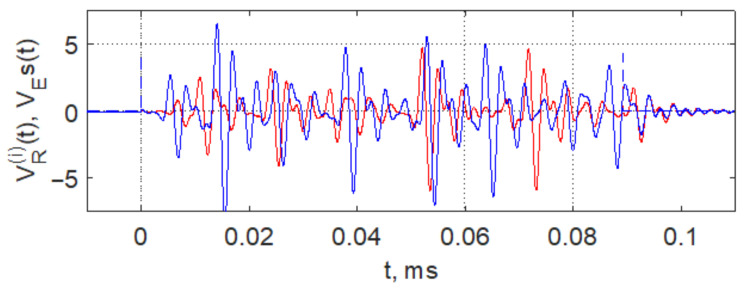
Forming pulses of different shapes by means of controlling the power supply voltage of chaos generator (1): $V_{E}^{\left(1\right)}=2.96$ V—red and $V_{E}^{\left(2\right)}=4.3$ V—blue.

The results of implementing this method (forming pulses by controlling the generator supply voltage) in numerical model (1) are shown in Figure 7, which shows the forms of radio pulses at the output $V_{R}$ of model generator (2) obtained for power supply video pulses of two different amplitudes. The duration of the video pulses is less than the duration of the coinciding (initial) section of the chaotic pulses. That is, the variation of the supply voltage amplitude leads to a change in the shape of the radio pulses.

### 3.3. Formation of Close-in-Shape Chaotic Radio Pulses in the Experimental Testbed

**Figure 8:**
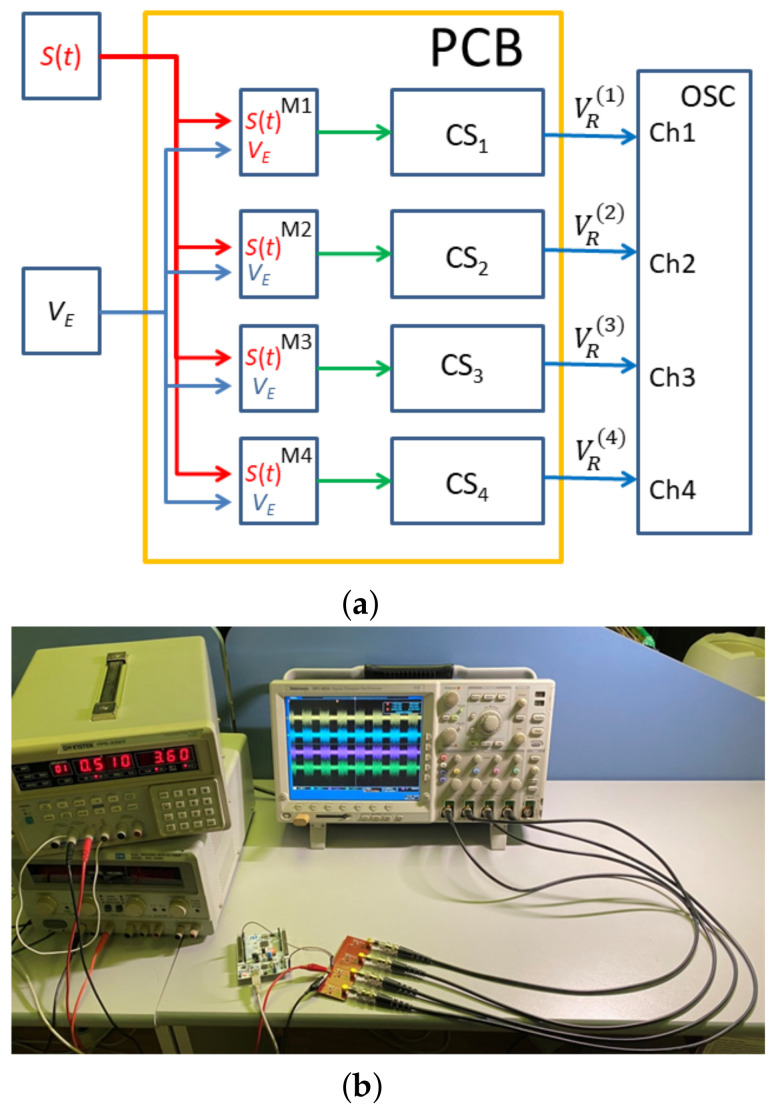
(**a**) The test stand layout: S(t) modulation signal, $V_{E}$ supply voltage, $M_{1}\ldots M_{4}$ power supply modulators, $CS_{1}\ldots CS_{2}$ chaos generators, $V_{R}^{\left(1\right)}$, $V_{R}^{\left(2\right)}$, $V_{R}^{\left(3\right)}$, $V_{R}^{\left(4\right)}$ output signals of the generators , OSC 4-channel oscilloscope; (**b**) experimental stand.

An experimental verification of the generation of chaotic radio pulses of similar shape and their reproduction by different instances of the generator was carried out on a test stand (Figure 8) in agreement with the scheme in Figure 8a. DC voltage $V_{E}$, supplied by a stabilized power source, was modulated by switches $M_{1}$, $M_{2}$, $M_{3}$, and $M_{4}$ under the control of signal s(t). Variation of supply voltage $V_{E}$ allowed us to change the oscillation mode of the generators.

**Figure 9:**
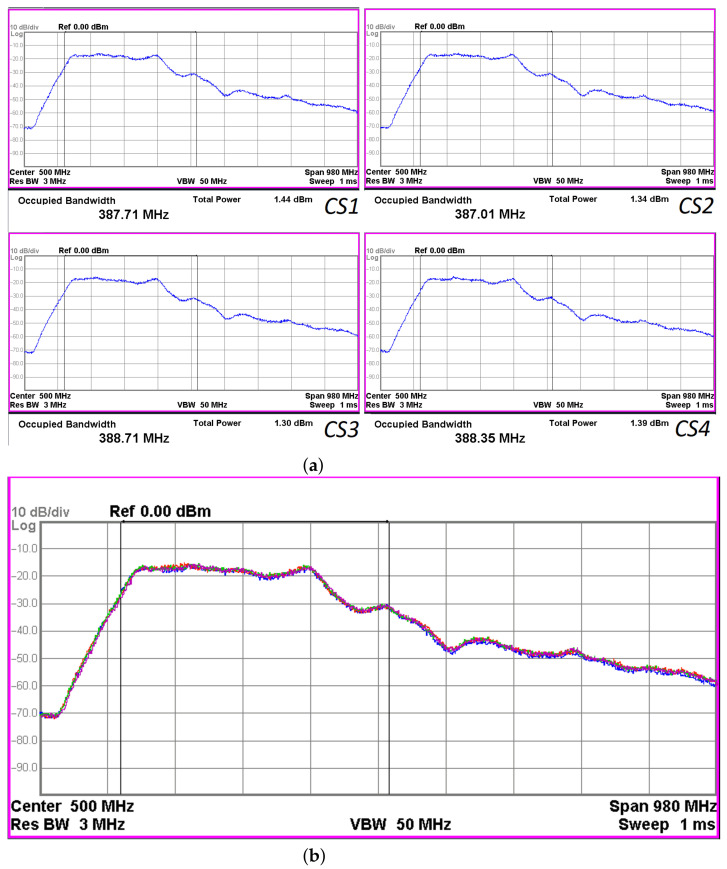
(**a**) Power spectra of the generator signals at supply voltage of $V_{E}=3.6$ V (spectrum analyzer Agilent N9000A): $CS_{1}$, $CS_{2}$, $CS_{3}$, $CS_{4}$; (**b**) spectra of the generator signals overlaid one over another: yellow—$CS_{1}$, green—$CS_{2}$, red—$CS_{3}$, blue—$CS_{4}$.

With a constant power supply, the generators operate in a continuous mode and form a chaotic signal with a power spectrum shown in Figure 9. The signal power of each generator is 1.4 mW at a supply voltage of 3.6 V and a current consumption of 50 mA. Direct comparison of the power spectra of the generator signals (Figure 9a) shows that they coincide with a high degree of accuracy (Figure 9b), which indicates good reproducibility of the oscillator modes.

**Figure 10:**
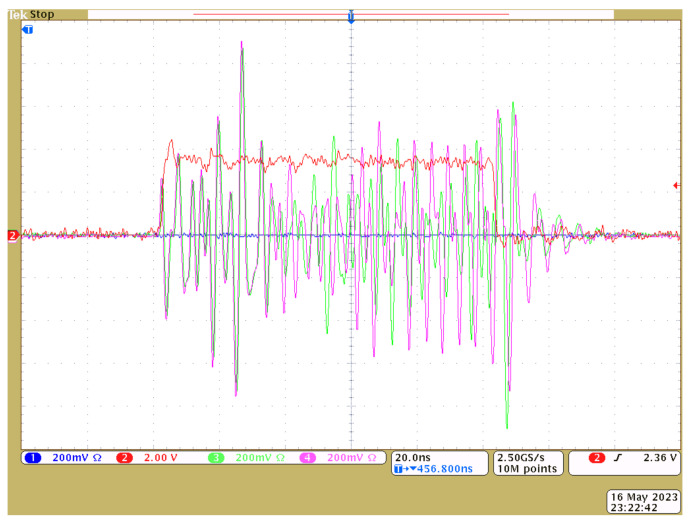
Oscilloscope screenshots with the waveforms of 100 ns chaotic radio pulses from two chaotic generators (green, magenta) and a video pulse of power supply (red) with the amplitude $V_{E}=3.6$ V.

An oscilloscope screenshot with the signals $V_{R}^{\left(1\right)}$, $V_{R}^{\left(2\right)}$ of generators $CS_{1}$, $CS_{2}$ is shown in Figure 10. The radio pulses of 100 ns duration, as well as a modulating video pulse, are shown. As can be seen, the shapes of the initial segments of the pulses coincide for about 30 ns.

To determine the degree of repeatability of the shape of the initial section of chaotic radio pulses generated by the testbed generators, these sections were directly compared by sampling the signals with an oscilloscope at a sampling rate of 2.5 GSpS. The oscilloscope memorized signals from the four generators (sequences of chaotic radio pulses). The radio pulses were extracted from each signal, and then they were superimposed on each other, with the starting moments of the radio pulses aligned.

**Figure 11:**
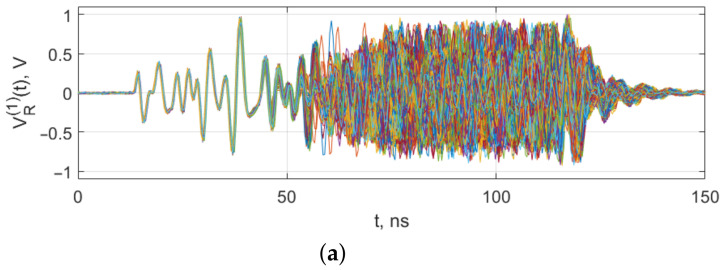

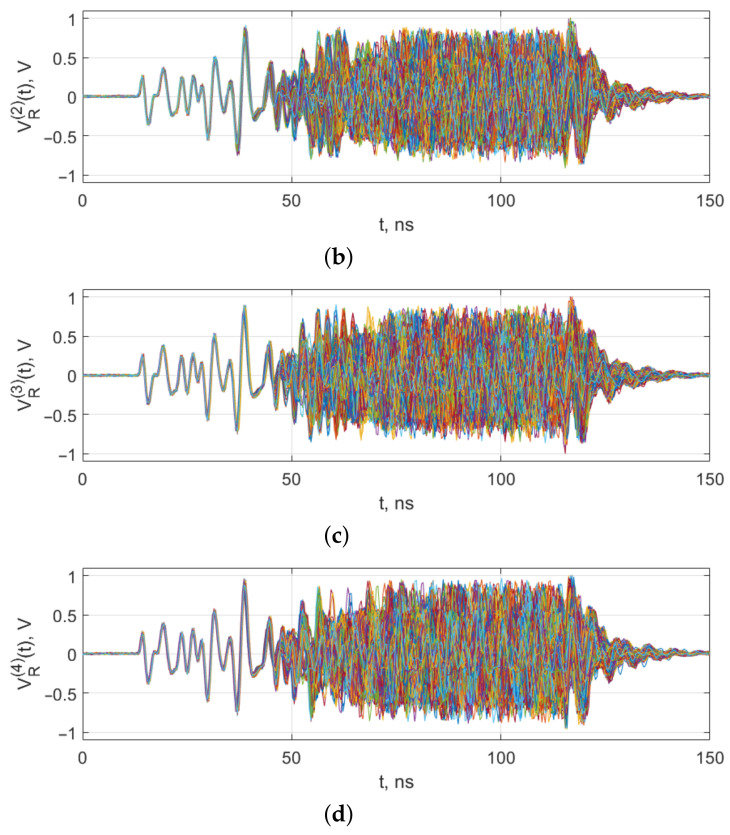
Shapes of the initial sections of 1000 pulses from generators CS1 (**a**), CS2 (**b**), CS3 (**c**), CS4 (**d**), power supply voltage $V_{E}=3.6$ V. Waveform of pulses are depicted by different colors, i.e., one colored line means one pulse.

The results of the comparison are given in Figure 11, which shows waveforms of 1000 radio pulses from each generator, aligned by their starting moments, at $V_{E}=3.6$ V. As can be seen, the initial pulse shapes coincide both in the signal from the same generator and in the signals generated by different instances of the generator. The duration of the coinciding segments was about 35 ns.

### 3.4. Generation of Different-Shape Pulses in the Experiment

The above experiments became the basis for the experiment on formation of pulses of various shapes, which can be reproduced both by the same generator instance and by different generator instances. As with the method of changing the pulse shape in the model, here the result is achieved by setting the amplitude of the power supply video pulses and taking the video pulse duration, which is the same as or less than the duration of the repeating initial section of the radio pulses.

**Figure 12:**
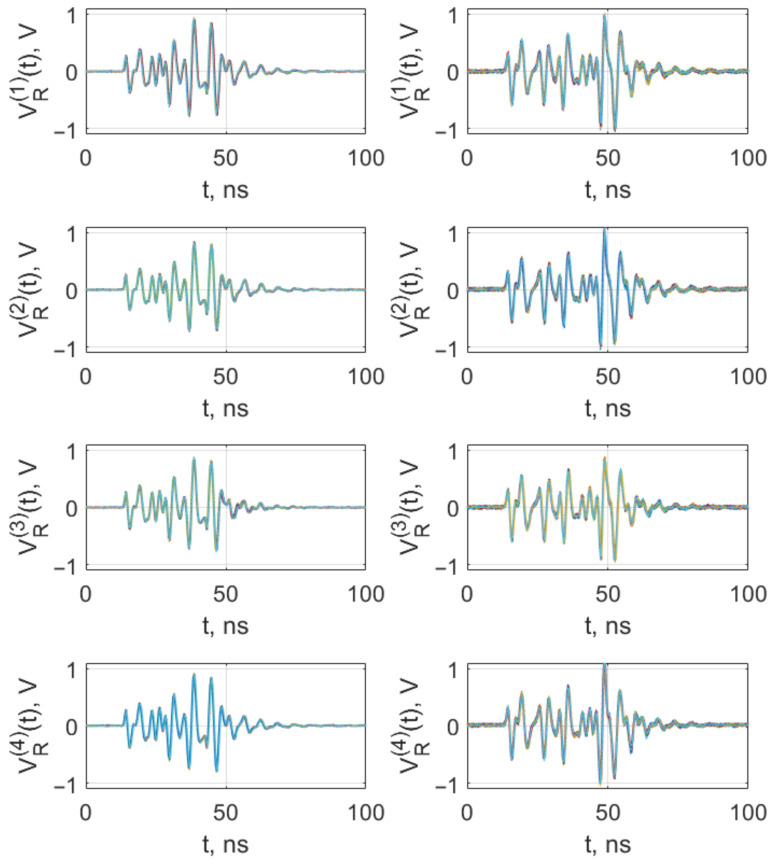
Shapes of the initial sections of 1000 pulses from generators CS1 ($V_{R}^{\left(1\right)}$), CS2 ($V_{R}^{\left(2\right)}$), CS3 ($V_{R}^{\left(3\right)}$), CS4 ($V_{R}^{\left(4\right)}$), power supply voltage $V_{E}=5.06$ V (**left column**), $V_{E}=3.6$ V (**right column**). Waveform of pulses are depicted by different colors, i.e., one colored line means one pulse.

In the experiment, the power inputs of the generators were fed with power supply pulses with a duration of 28 ns and amplitudes 3.6 V and 5.06 V. As a result, pulses of two different shapes were obtained, fully reproducible from pulse to pulse, both by one generator and by different instances of the generator (Figure 12).

Thus, by controlling the power supply voltage of chaos generators with a certain duration of video pulses, one can repeat (reproduce) the shape of the pulses and change this shape by changing the amplitude of the power supply video pulses.

## 4. Discussion

The idea of using the properties of chaotic dynamic systems to form and to modulate signals of various shapes for wireless communication problems has been discussed in scientific circles since the beginning of the application of chaotic oscillations in radio engineering. However, these ideas were theoretical in one way or another and were seldom brought to the stage of physical implementation (some of the papers in this area are listed in the introduction), that be able to compete practically with existing technical solutions.

When talking about chaotic signals generated by digital systems, one can hardly avoid comparing their characteristics with those of regular digital signals intended for spectrum spreading or for implementing processing gain reception (Walsh functions, m-sequences, Gold codes, Barker codes, and Kasami codes), which are well-known to specialists and have the required signal properties. Therefore, direct opposition of digital systems generating chaotic signals to digital systems of conventional noise-like signals does not always reveal the advantages of the former.

In this regard, the analog method for forming signals with a non-repeating (irregular) shape in the microwave range has a natural advantage over digital generation methods, since such signals do not require transfer to the RF range. The shape of such signals depends only on the supply voltage due to the sensitivity of the chaotic trajectory to the initial conditions or to perturbations in the course of the trajectory evolution. This gives us a natural radio engineering way of forming signals of complex shape with the ability to control this shape and reproduce its characteristics.

The generation method proposed in this paper is of interest in simple analog devices, in which it is not advisable to use complex digital processing and digital signal synthesis (e.g., active or passive RFID, simple wireless sensors) but in which it is necessary to generate a complex-shape signal in order to organize processing gain and coherent reception. The ability to reproduce the signal shape directly in the microwave range creates the prerequisites for the development of transceiver systems based on the coherent addition of signals at the receiving point (due to their time-coherent emission) and subsequent coherent processing of these signals in the receiver. Controlling the signal shape by varying a single parameter (supply voltage) means the possibility of coherent emission from several sources, which can be interesting in the context of creating beam-forming systems using chaos generators for various applications. Based on the experimental results, we can conclude that coherent processing (emission and reception) is also available for UWB signals produced by chaotic oscillation systems.

Chaos generators can play the role of universal signal sources, with which UWB microwave signals of various shapes can be obtained. Such signals are formed directly in the required frequency range, without the need for additional operations of transferring them to the required frequency band. This is extremely useful when creating sources of complex shape oscillations in both the microwave range and in the millimeter range of next-generation wireless solutions because the basic principles of the chaotic oscillation formation are preserved.

## 5. Conclusions

The purpose of this paper was to demonstrate the fundamental ability of synthesizing microwave oscillations of the same shape using different analog chaotic generators. The efficiency of the method has been experimentally verified on the example of four generators controlled by a single power source. The possibility of generating identical radio pulses with a duration of 10–15 quasi-periods of oscillations was shown, demonstrating that it is possible to control the shape of these pulses.

Demonstration of this fundamental ability opens the way for further research in the direction of controlled complication of the oscillation form (due to the sensitivity of the chaotic trajectory shape to the modulation voltage) and in the direction of increasing the length of the trajectory that can be reproduced by different generator instances. In this paper, this question remains open, but the properties of the system demonstrated here provide a basis for this.

From a theoretical point of view, an increase in the duration of reproducible (repeated) sections is necessary to increase the signal dimension of the chaotic radio-pulse $B=2\Delta fT_{P}$, which will allow us to have large processing gain (accumulation).

Another group of theoretical issues to be studied is cross- and autocorrelation properties of radio pulses generated in this way. Obviously, the proposed method will likely not provide a strictly orthogonal set of impulse signals, as is done when generating digital noise-like sequences with preassigned correlation properties. At the same time, from the point of view of the current state of wireless communications, this cannot be considered a serious limitation of this method. In the field of wireless communication, there is a large trend towards the development of non-orthogonal media access methods (the so-called NOMA methods), which are in demand in the field of mass deployment of transceivers. It is practically impossible to provide complete mutual orthogonality in the media, but such transceivers must be able to generate signals of various shapes.

Of course, it will also be necessary to conduct research related to various aspects of implementation of the proposed approach, which will determine the possibilities and limits of this approach as well as its effectiveness in various applied problems. For example, if we talk about coherent reception of pulsed signals by reproducing a copy of the signal in the receiver using the proposed method, then it will be necessary to develop specific reception schemes, circuit designs, and signal processing methods. Additionally, the reproducibility of the shape and characteristics of chaotic radio pulses when they are generated by physically separate instances of generators located in different devices (transmitter and receiver) will also be the subject of future research.

If we raise the question of beam-forming using spaced-apart UWB radio pulse generators, then it will be necessary to work out methods for generating identical pulses by physically separated device instances loaded on their own antennas, which will be controlled by independent modulation signals. It will also be necessary to investigate the propagation effect of identical pulses in a wireless communication channel on their characteristics and on the use of them in beam-forming.

Summarizing the above, further efforts will be focused on the research and development of :

- Methods for increasing the duration of the reproducible section of chaotic radio pulses in order to increase their base;
- Auto- and cross-correlation properties of such signals;
- Practical methods for coherent processing of pulses in the receiver using the proposed method;
- Methods for generating such pulses using spaced-apart devices;
- Issues related to the emission of pulses and the effect of the emitting circuits on the pulse shape;
- Questions of beam-forming in real communication channels using such pulses.

## Data Availability

Not applicable.

## Abbreviations

The following abbreviations are used in this manuscript:

DCC	Direct Chaotic Communication
FPGA	Field-Programmable Gate Array
RFID	Radio-Frequency Identification
UWB	Ultra-Wideband
USP	Ultra-Short Pulses

## Appendix A. Symbol Definitions

Symbol definitions used in this manuscript are enumerated in Table A1.

**Table A1:** The symbol definitions.

Symbol	Definition
$C_{0},C_{1},C_{2},C_{3},C_{4},C_{5},C_{6}$	Values of capacitors in Figure 1
$L_{1},L_{2},L_{3},L_{4},L_{5}$	Values of inductance in Figure 1
$R_{E},R$	Values of resistors in Figure 1
$R_{1},R_{2},R_{3},R_{4},R_{5}$	Values of the resistances of inductances $L_{1}\ldots L_{5}$, respectively
$V_{0},V_{1},V_{2},V_{3},V_{4},V_{5},V_{6}$	Dimensional voltages over capacitors $C_{0}\ldots C_{6}$, respectively
$V_{a},V_{R}$	Dimensional voltages in point A and over the load resistor *R*, respectively
$J_{1},J_{2},J_{3},J_{4},J_{5}$	Dimensional currents through inductances $L_{1}\ldots L_{5}$, respectively
$V_{L1}$, $V_{L2}$, $V_{L3}$, $V_{L4}$, $V_{L5}$	Voltages across the inductances $L_{1}$, $L_{2}$, $L_{3}$, $L_{4}$, $L_{5}$, respectively
*t*	Dimensional time
$t_{d}$	Nondimensional time
$v_{0},v_{1},v_{2},v_{3},v_{4},v_{5},v_{6}$	Nondimensional voltages over capacitors $C_{0}\ldots C_{1}$, respectively
$v_{a},v_{R}$	Nondimensional voltages in point *A* and over the load resistor *R*, respectively
$i_{1},i_{2},i_{3},i_{4},i_{5}$	Nondimensional currents through inductances $L_{1}\ldots L_{5}$, respectively
$\alpha =2\pi \sqrt{C_{5}L_{5}}$	Dimensionless coefficient
$V_{T}$	Barrier potential of the transistor
$J_{C}$	Dimensional current through the collector
$j_{C}$	Nondimensional current through the collector
$I_{0}$	Reverse saturation current of the base–emitter junction (dimensional)
$i_{0}$	Reverse saturation current of the base–emitter junction (nondimensional)
β	Static forward current transfer ratio
$V_{n}$	Thermal voltage
$V_{E}$	Dimensional power supply voltage
s(t)	Sequence of rectangular video pulses with unit amplitude (nondimensional)
*M*	Modulator (switch)
$V_{R}$	Output signal loaded on resistor *R*
$v_{0}^{\left(0\right)},v_{1}^{\left(0\right)},v_{2}^{\left(0\right)},v_{3}^{\left(0\right)},v_{4}^{\left(0\right)},v_{a}^{\left(0\right)},v_{R}^{\left(0\right)}$	Nondimensional voltage values corresponding to the equilibrium state
$i_{1}^{\left(0\right)},i_{2}^{\left(0\right)},i_{3}^{\left(0\right)},i_{4}^{\left(0\right)},i_{5}^{\left(0\right)}$	Nondimensional current values through inductances $L_{1}\ldots L_{5}$ corresponding to the equilibrium state
$V_{R}^{\left(1\right)}$, $V_{R}^{\left(2\right)}$, $V_{R}^{\left(3\right)}$, $V_{R}^{\left(4\right)}$	Output signals of the generators on the testbed
$CS_{1},CS_{2},CS_{3},CS_{4}$	Chaotic oscillators of the testbed
Δf	Frequency bandwidth of chaotic radio-pulses
$T_{P}$	Chaotic pulse length

## Appendix B. Equations of the Dynamic System

The dynamic system of the circuit in Figure 1 consists of 12 equations, i.e., 7 equations for currents and 5 equations for voltages.

Below, the equations are described briefly in the same order as in (1):

1. Equation for voltage at the point *A* as the derivative of the current $J_{5}$ through inductance $L_{5}$.
2. Equation for the sub-circuit $C_{0}-L_{1}-C_{4}$: the voltage across inductance $L_{1}$ is the difference between the voltages over capacitors $C_{4}$ and $C_{0}$.
3. Equation for the currents in the sub-circuit $L_{1}-C_{0}-BE\;junction$: $J_{1}$ is the sum of the currents through $C_{0}$ and the base–emitter junction.
4. Equation for the currents in the sub-circuit $L_{2}-C_{1}-CE\;junction$: $J_{2}$ is the sum of the currents through capacitor $C_{1}$ and the collector–emitter junction.
5. Equation for the currents in the sub-circuit $L_{3}-C_{2}-L_{2}$: $J_{3}$ is the sum of the currents through capacitor $C_{2}$ and $J_{2}$.
6. Equation for the currents in the sub-circuit $L_{4}-C_{3}-L_{3}$: $J_{4}$ is the sum of the currents through $C_{3}$ and $J_{3}$.
7. Equation for the sub-circuit $C_{2}-L_{2}-C_{1}$: the voltage across inductance $L_{2}$ is $V_{2}=V_{L2}+V_{1}$.
8. Equation for the sub-circuit $C_{3}-L_{3}-C_{2}$: voltage across inductance $L_{3}$ is $V_{L3}=V_{3}-V_{2}$.
9. Equation for the sub-circuit $C_{4}-L_{4}-C_{3}$: the voltage across inductance $L_{4}$ is $V_{4}=V_{L4}+V_{3}$.
10. Equation for the currents in the sub-circuit $C_{4}-L_{1}-R_{E}-L_{4}-C_{5}$: the current through capacitor $C_{4}$ is the sum of currents $J_{1}$ and $J_{4}$, through resistor $R_{E}$ and capacitor $C_{5}$.
11. Equation for the currents in the sub-circuit $C_{5}-L_{5}-C_{6}$: the current through capacitor $C_{5}$ is the sum of current $J_{5}$ and the current through capacitor $C_{6}$.
12. Equation for the currents in the sub-circuit $C_{6}-R$: the current through capacitor $C_{6}$ is equal to the current through load resistor *R*.
